# Berlin's medical students' smoking habits, knowledge about smoking and attitudes toward smoking cessation counseling

**DOI:** 10.1186/1745-6673-5-9

**Published:** 2010-04-16

**Authors:** Bianca Kusma, David Quarcoo, Karin Vitzthum, Tobias Welte, Stefanie Mache, Andreas Meyer-Falcke, David A Groneberg, Tobias Raupach

**Affiliations:** 1 Institute of Occupational Medicine, Charité School of Medicine, Free University and Humboldt University, Thielallee 69-73, 14195 Berlin, Germany; 2 Department of Respiratory Medicine, Hannover Medical School, Carl-Neuberg-Straße 1, 30625 Hannover, Germany; 3 Strategy Centre for Health, Health Care Campus North Rhine Westphalia, Universitätsstraße 136, 44799 Bochum, Germany; 4 Department of Cardiology and Pneumology, University Hospital Göttingen, Germany

## Abstract

**Background:**

Diseases associated with smoking are a foremost cause of premature death in the world, both in developed and developing countries. Eliminating smoking can do more to improve health and prolong life than any other measure in the field of preventive medicine. Today's medical students will play a prominent role in future efforts to prevent and control tobacco use.

**Methods:**

A cross-sectional, self-administered, anonymous survey of fifth-year medical students in Berlin, Germany was conducted in November 2007. The study explored the prevalence of smoking among medical students. We assessed their current knowledge regarding tobacco dependence and the effectiveness of smoking cessation methods. Students' perceived competence to counsel smokers and promote smoking cessation treatments was also explored. Analyses were based on responses from 258 students (86.6% response rate).

**Results:**

One quarter of the medical students surveyed were current smokers. The smoking rate was 22.1% among women, 32.4% among men. Students underestimated smoking-related mortality and the negative effect of smoking on longevity. A considerable number of subjects erroneously assumed that nicotine causes coronary artery disease. Students' overall knowledge of the effectiveness of smoking cessation methods was inadequate. Only one third of the students indicated that they felt qualified to counsel patients about tobacco dependence.

**Conclusions:**

This study reveals serious deficiencies in knowledge and counseling skills among medical students in our sample. The curriculum of every medical school should include a tobacco module. Thus, by providing comprehensive training in nicotine dependence interventions to medical students, smokers will have access to the professional expertise they need to quit smoking.

## Background

Smoking is the leading cause of preventable morbidity and mortality in the world [1]. Tobacco use claims worldwide 5.4 million lives each year [2]. Although overall cigarette consumption has declined for decades in high-income countries, smoking rates are on the rise in low- and middle-income countries [3]. The negative health consequences of smoking are considerable and include cancers of the lung and other organs, chronic lung disease, stroke and other cardiovascular disease [4-6]. Smoking during pregnancy can lead to spontaneous abortions, low birth weight, and sudden infant death syndrome [7]. Exposure to secondhand smoke also has serious health effects [8,9].

The benefits of smoking cessation have been well demonstrated. Smoking cessation reduces health risks and improves quality of life. The cumulative risk of dying of cardiovascular and lung diseases can be drastically reduced (up to 90%) if smokers quit smoking, even late in life [10,11]. Therefore, every smoker should be actively encouraged to give up smoking. Due to tobacco's highly addictive properties, cessation attempts need to be supported by health care professionals to achieve long-term abstinence.

Physicians are in an ideal position to advise and educate patients about the dangers of smoking. Moreover, they act as visible role models and may unintentionally affect the smoking behavior of others [12]. Their own smoking habits may cloud their judgement and influence their ability to adequately counsel smokers. They are also more likely to maintain attitudes that prevent them from providing patients with anti-smoking advice [13,14]. As one can assume many of their personal smoking behaviors and beliefs are formed during their medical education, any successful tobacco control measures within the medical profession will need to begin prior to graduation from medical school. Undergraduate curricula must include teaching modules focusing on the responsibility that doctors have in disease prevention and training in specific smoking cessation techniques.

Despite the responsibility that physicians have to their smoking patients, research suggests medical students still do not receive adequate training. A worldwide survey of tobacco curricula conducted ten years ago revealed that only 11% of medical schools had devoted specific teaching time to tobacco and smoking cessation [15]. Furthermore, a series of significant international studies reported serious deficiencies in medical education on smoking-related issues. Relatively few students (15-38%) found it necessary to advise smokers to quit before they had developed a smoking-related disease [16-19].

In a recent study, Raupach and colleagues [20] assessed the knowledge of medical students from two European cities: London (UK) and Göttingen (Germany). Medical students at both study sites lacked relevant information about smoking and its consequences for patients' health. Students underestimated smoking-related mortality and overestimated the chances of reaching old age as a smoker. Furthermore subjects' knowledge of the effectiveness of smoking cessation methods was deficient. Less than a third of medical students felt able to counsel smoking patients. The authors concluded that current curricula about tobacco dependence and control in medical schools need to be improved.

The purpose of the present study is to review current knowledge of students in their penultimate year of medical school regarding tobacco dependence, smoking-related mortality and the effectiveness of various smoking cessation techniques with regard to the study by Raupach and colleagues [20]. We also explored students' perceived competence to counsel smokers and promote smoking cessation treatments. Furthermore, smoking rates among the participating medical students were determined and students' smoking behavior was correlated to their attitudes toward counseling of smoking patients. Aim of the present study is to increase external validity of the study by Raupach and colleagues. by replication of the findings in another sample.

## Methods

### Study population and study design

A cross-sectional survey was conducted at Charité medical school (Berlin, Germany) in No-vember 2007. Fifth-year medical students attending a required course in occupational medicine were included in this study. The curriculum in Berlin has no specific tobacco teaching module; however, tobacco-related issues are addressed in organ-specific modules.

The Charité ethics committee granted approval for the current study prior to the survey. Participation in the study was voluntary and informed consent was implied if students completed and returned their questionnaire.

### Measures

The questionnaire used in this study was developed and piloted by Raupach and colleagues [20]. Items on the questionnaire pertained to students' demographic characteristics, such as gender and age, smoking status and knowledge about smoking and the effectiveness of several smoking cessation methods.

Smokers were distinguished from nonsmokers by the question: "Do you smoke cigarettes at all nowadays?" For the purpose of this study, the broad definition of tobacco use included both daily and occasional tobacco use. The extent of students' smoking behavior was assessed in further questions about the number of cigarettes smoked per day, prior smoking history (including relapse) and the age at the onset of smoking. A Fagerström test for nicotine dependence (FTND) was calculated for all current smokers [21].

An inventory of questions was used to assess students' knowledge about smoking-related morbidity and mortality and the effectiveness of various smoking cessation treatments.

Students answered three open response questions: "Please estimate how many people die of smoking-related diseases annually in Germany"; "In your opinion, what components of tobacco smoke are responsible for smokers' increased risk of coronary artery disease?" and "Please estimate: What percentage of smokers in developed countries eventually die of smoking-related diseases?"

Additionally, students were asked to estimate smoking-attributable fractions (i.e. the percentage of all cases of a specific disease caused by smoking) for different types of diseases using an 11-point scale. According to the U.S. Surgeon General [22] and Bresnitz [23], the smoking-attributable fraction of chronic obstructive lung disease (COPD) is approximately 80-90%. In addition, 85-90% of all lung cancers are attributed to smoking [24,25]. Therefore, for these diseases, ratings of 80% or 90% were regarded as correct.

Students estimated the effectiveness of different smoking cessation methods (willpower alone, advice from a general practitioner, nicotine replacement therapy (NRT), cessation program, self-help material, and acupuncture) on a four-point Likert scale, ranging from "hardly effective" to "very effective". Smoking cessation was considered to be very effective if the continuous abstinence rate was at least 30% after 1 year. As in the British Doctors' Study [26], participating students were also asked to indicate whether they personally knew smokers and nonsmokers who lived to the age of 90.

Finally, students rated their competence in supporting their patients' cessation attempts. They were asked whether they felt "competent to counsel a smoker who is seeking help in order to give up smoking".

### Data collection

Questionnaires were handed out at the beginning of the seminar and collected during the same session. In total, 258 of the 298 students who received questionnaires returned them (response rate 86.6%). Incomplete questionnaires were included if data on students' smoking status and/or assessment of smoking-related health risks and the effectiveness of smoking cessation methods were available.

### Statistical analysis

The number of participants' responses used in the discrete statistical analyses varied due to missing data for certain variables. Frequency distributions were used to describe respondents' demographic characteristics, smoking behaviors, and other variables. Gender differences in smoking rates were investigated using the Chi-square tests for categorical variables. Statistically significant differences in knowledge with respect to students' age were evaluated by one-way analysis of variance (ANOVA). Chi-square tests were conducted to examine the association of the dependent variables (knowledge) and independent variables (such as smoking status and demographic variables). To avoid type II errors, Φ was used instead of the chi-square distribution if the expected frequencies were too low (more than 20% of the cells had an expected count less than 5). Analyses were performed using SPSS version 17.0.

## Results

### Participants

The majority of participants were female (70.2%). The mean age of participants was 26.4 years (SD = 3.86 years, range 22-48 years). An ANOVA comparison of students' overall age by gender revealed no significant age difference between male and female medical students (F = 1.55, *df *= 1, *p *= .214). Further information about participants' demographic characteristics and prevalence of smoking is presented in Table 1.

**Table 1:** Participants' demographic characteristics and smoking behaviors

	Women	Men	Total
**Age (years)**			
Median	25	25	25
Range	22-48	22-44	22-48
			
**Smoking status**			
Total N(%) of past/current smokers	79 (43.6%)	31 (40.3%)	110 (38.8%)
Total N (%) of current smokers	40 (22.1%)	25 (32.4%)	65 (25.2%)
N (%) of current smokers wanting to quit	28 (70%)	13 (52%)	41 (63.1%)
N (%) current smokers Had a relapse before	27 (67.5%)	15 (60%)	42 (64.6%)

Roughly one quarter of the participating medical students were smokers. The prevalence of tobacco use was 22.1% among women and 32.4% among men. A further 18.6% were ex-smokers. Current smoking status did not vary significantly between genders (χ^2 ^= 2.83, *df *= 1, *p *= .123). The number of cigarettes smoked per day was not significantly different between male and female students (χ^2 ^= 5.67, *df *= 3, *p *= .132). Seventy-nine percent of participants smoked one to ten cigarettes/day.

Over 60% of smokers indicated that they wished to stop smoking and 54% had tried to quit for ≥ 24 hours at least once. When classified according to the Fagerström test for nicotine dependence (FTND [21]), the overwhelming majority (90.8%) were light smokers (Table 2).

**Table 2:** Daily smoking habits, habit duration in years, FTND, and mean values

Variable	(%)
**Daily number of cigarettes (cigarettes/day)**	
1-10	78.5
11-20	12.3
21-30	9.2
	
**Smoking duration (years)**	
1-5	13.3
6-10	41.7
11-15	33.3
> 16	11.7
	
**FTND†**	
Light smoker	90.8
Moderate smoker	3.1
Heavy smoker	6.1
	
**Mean values (SD)**	
Age at first tobacco use‡	16.7 (2.98)
Years of smoking*	10.4 (4.36)

† Fagerström Test for Nicotine Dependence (FTND); ‡ based on current and previous smoke-rs (N = 108); * Based on current smokers (N = 60)

### Smoking-attributable morbidity and mortality

**Figure 1:**
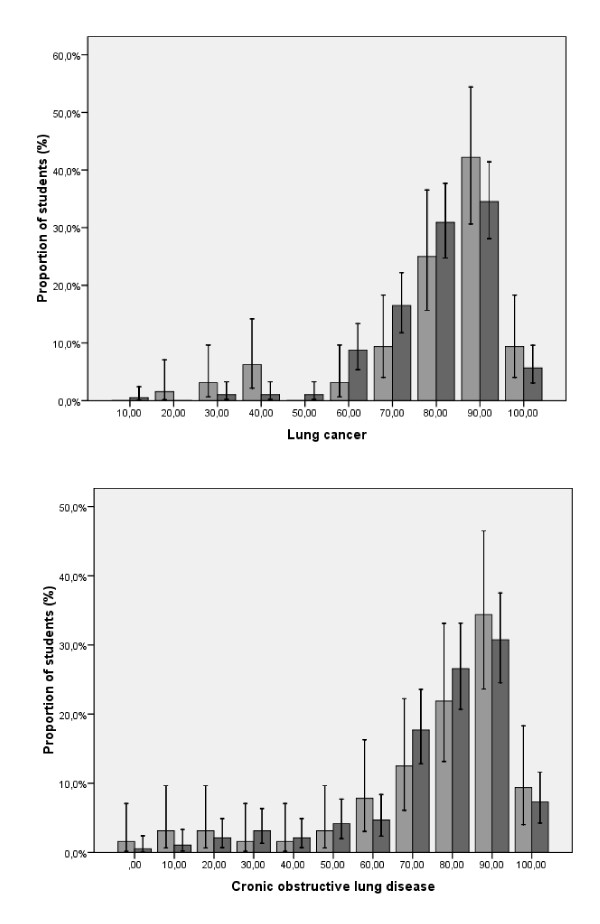
**Smoking-attributable fractions of lung cancer and chronic obstructive pulmonary disease as estimated by medical students depending on smoking status, light grey bars, smokers; dark grey bars, nonsmokers**. Error bars indicate 95% *CI*s.

Figure 1 shows students' estimates of smoking-attributable fractions as a function of smoking status for lung cancer and chronic obstructive lung disease. These findings reveal that the majority of medical students correctly identified rates of smoking-attributable lung cancer and COPD. However, at least one-fifth of the surveyed students believed that smoking was responsible for fewer than 70% of COPD cases.

According to John and Hanke [27] annual smoking-related mortality rate ranges from 130.000 to 150.000 in Germany. Only 3.2% of students provided an estimate from within this range. The median of students estimated death rates attributable to tobacco smoking was 100.000. As in the Göttingen sample [20] Berlin medical students underestimated smoking-related mortality (see table 3).

**Table 3:** Students' estimates of annual smoking-related mortality rates in Germany

	Nonsmoker (n = 194)	Smoker (n = 64)
Estimate (deaths per year)	N (%)	N (%)
0-100.000	98 (50.5)	35 (54.7)
100.001-200.000	14 (7.2)	9 (14.1)
200.001-300.000	17 (8.8)	3 (4.7)
300.001-400.000	7 (3.6)	4 (6.3)
400.001-500.000	16 (8.2)	1 (1.6)
> 500.000	16 (8.2)	2 (3.1)
Missing values	26 (13.4)	10 (15.6)

The overall findings from the British Doctors' Study reveal that between half and two-thirds of smokers who smoke at least 20 cigarettes/day will ultimately die from a smoking-related disease. Roughly one quarter of students in this study also gave an answer within this range. More smokers (47.3%) underestimated this number than nonsmokers did (41.1%) (φ = .41, *p *< .03).

### Tobacco toxins

**Figure 2:**
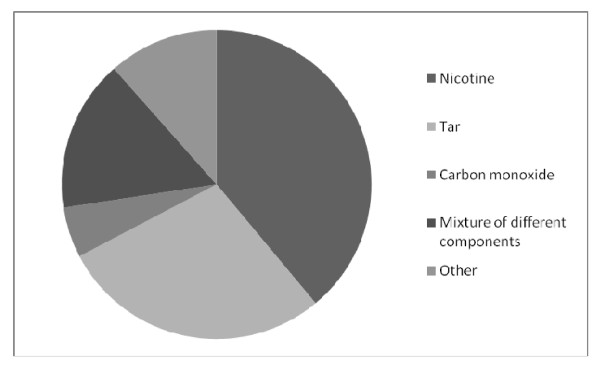
**Students' answers to question what components of tobacco smoke are responsible for smokers' increased risk of coronary artery disease**.

Nicotine, tar, carbon monoxide, and a mixture of different components were toxic agents mentioned by at least 5% of medical students when asked which tobacco component causes coronary artery disease (Figure 2). Thirty-nine percent of students blamed nicotine alone for coronary artery disease and 28.37% found tar to be solely responsible for its etiology.

### Effectiveness of tobacco cessation method

**Figure 3:**
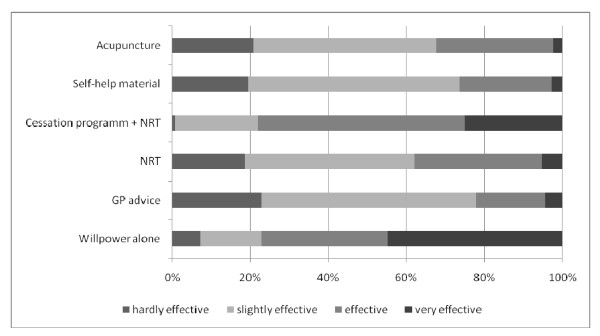
**Students' perceptions oft he long-term effectiveness of different approaches to smoking cessation**. Students rated effectiveness on a 4-point Likert scale with high effectiveness defined as a continuous abstinence rate of 30% after 1 year. GP, general practitioner.

Students assigned the highest effectiveness to "willpower alone", thus rating it above behavioral support programs plus NRT or NRT alone. Advice from a general practitioner scored similarly to self-help material and acupuncture (Figure 3). Further analysis revealed no differences in the perceived effectiveness of various cessation methods according to students' smoking status, except behavioral support programs - nonsmokers rated their effectiveness higher than smokers (χ^2 ^= 12.19, *df *= 3, *p *< .01).

### Smoking and life expectancy

**Figure 4:**
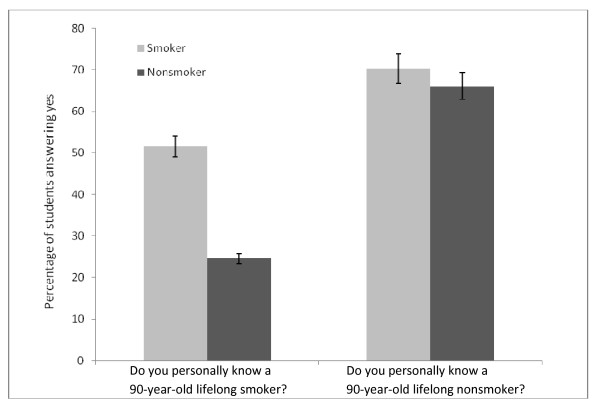
**Students' answers to questions regarding the chances of reaching old age depending on smoking status**.

Students were asked whether they personally knew smokers and nonsmokers who lived to be at least 90 years old to assess students' perceptions of the effect of smoking on longevity. A significantly greater percentage of smokers than nonsmokers stated that they personally knew lifelong smokers reaching old age (χ^2 ^= 16.18, *df *= 1, *p *< .000). The proportion of students indicating that they knew a 90-year-old nonsmoker was equal in both groups (χ^2 ^= .38, *df *= 1, *p *= .539; Figure 4).

### Perceived competence to counsel smokers

Although almost all students (96.1%) stated that every smoker should be advised to stop smoking, only half (51.2%) of them reported actually having recommended smoking cessation to a patient. Only one third of students indicated that they felt qualified to counsel patients about tobacco dependence. Further analysis revealed that non-smokers (75.7%) were particularly unsure of their counseling skills compared with smokers (50.8%) and ex-smokers (57.4%) (χ^2 ^= 14.08, *df *= 2, *p *< .01). Female non-smokers rated their competence in tobacco cessation counseling significantly lower than their male colleagues (19.6% vs. 35.7%; *p *< .05). No significant gender differences were found between self-assessed competencies of smokers (45% vs. 56.5%; *p *= .38) and ex-smokers (46.2% vs. 25%; *p *= .27).

## Discussion/Conclusion

The aim of this study was to evaluate Berlin's medical students' smoking habits, knowledge about smoking and attitudes toward smoking cessation counseling. Our investigation found several important results which are worth to discuss.

One quarter of all medical students surveyed in our study were current smokers, a rate similar to that of the general population [28]. It seems that medical students' undergraduate education about the hazards of smoking have relatively little impact on smoking behavior [29]. Various cross-sectional investigations have suggested that there is an alarming worldwide trend for smoking rates to increase during students' time at medical schools [29-34]. To discourage smoking among medical students, it is essential to introduce teaching on tobacco dependence and cessation early in the course of medical school. Tobacco curriculum should continue throughout the entire medical degree as it is difficult to determine whether this effect directly reflects students' seniority, age or both [35]. Contrary to findings from other studies of medical students, the current smoking status did not vary significantly between genders [36]. Moreover, smoking prevalence among women was higher in the present study than reported by most studies in other countries [17,37]. However, these findings are consistent with other studies conducted in German medical schools [38,39].

Although the majority of medical students correctly identified rates of smoking-attributable lung cancer and COPD, they lacked sufficient knowledge about tobacco and its effects. At least one-fifth of the participants underestimated the rate of smoking-related COPD. Furthermore, students in our study, as in the Göttingen sample [20], greatly underestimated smoking-related mortality and disease - smokers gave significantly less appropriate answers than non-smokers. This finding is consistent with other studies in this field [16,17]. Underestimation of smoking-attributable morbidity and mortality could have a negative impact on medical students' efforts to counsel smoking patients in the future. The belief that smoking is not life-threatening or, at least, not too hazardous might undermine future physicians' promotion of smoking cessation.

Of all the harmful substances contained within tobacco smoke, a large proportion of students in our study believed that nicotine alone is responsible for coronary artery disease (Figure 2). These results are consistent with the study by Raupach and colleagues [20] and of particular interest given that many of German medical textbooks use misleading terms for the health effects of smoking [40]. The words "smoking" and "nicotine" are used synonymously within the context of cardiovascular risk factors. This misuse [41] erroneously suggests a casual relationship between nicotine and coronary heart disease and may explain German general practitioners' hesitancy to recommend NRT.

Students in our study knew little about cessation techniques. As in the Göttingen sample [20], subjects rated "willpower alone" as the most effective of all tobacco cessation methods, rating it above NRT alone or cognitive behavioral support programs plus NRT, although the former has been shown to be effective and safe [42] and the latter has demonstrated optimal cessation outcomes [42-44]. Moreover, students rated advice from a general practitioner similarly to self-help material and acupuncture, despite the fact that evidence does not support the efficacy of acupuncture as a smoking cessation treatment [43,45] and research has shown that GP consultations with patients yield one-year cessation rates of 3-10% [42,46]. This underestimation of physicians' ability to promote smoking cessation may adversely affect their professional practice later in life. Future general practitioners who attach little importance to physicians' advice are unlikely to make an effort to provide smoking-prevention counseling once they have become general practitioners themselves [47]. The finding that smokers and non-smokers assess the effectiveness of cessation methods differently [48] could be replicated to a certain extent in our study. Reasons for this may lie in subjects' different levels of education in the two studies (academic versus non-academic sample).

Apart from knowledge about effectiveness of different cessation methods and smoking-related morbidity and mortality, a person's experience and smoking status may have an influence on the counseling of smoking patients. In our sample, students' perceptions of the effect of smoking on longevity differed with respect to their personal smoking habits. These results are consistent with the study by Raupach and colleagues [20]. The results of the British Doctors' Study [26] indicate that a nonsmoker's chance of living to the age of 90 years (24%) is six times greater than that observed in smokers (4%). The two questionnaire items related to this study assessed students' personal experiences rather than their knowledge about smokers' and nonsmokers' life expectancies. More smokers than nonsmokers in our sample stated that they personally knew lifelong smokers (Figure 4). Research suggests that the smoking habits of parents may have an influence on whether or not a medical students smokes [34,39,49]. Coming from families or communities with higher smoking prevalence could increase one's chances of personally knowing a 90-year-old lifelong smoker. In addition, cognitive dissonance may also play a role for smokers [50]. However, it may also reflect an excessively optimistic view of smoking held by smoking medical students, which might eventually undermine their own commitment to promoting smoking cessation among their patients.

A large proportion of students thought that they did not have adequate skills to counsel patients about smoking. In fact, only half of them reported actually having recommended smoking cessation to a patient, possibly due to a perceived lack of competence pertaining to clinical behavior. Similar trends have been found among practicing doctors. Although 70% of smokers visit a general practitioner annually, most are not advised or assisted in smoking cessation matters [51]. Differences found between perceived counseling skills of smokers and non-smokers were surprising because research suggests that more nonsmokers than smokers are active in smoking cessation counseling [52-55]. This difference may be due to the addictive nature of smoking. Smokers may feel more apt to put themselves in a smoking patients' position than non-smokers. A large number of smokers in our study indicated that they wished to stop smoking and about half of them had made one or more quit attempts. Further research is needed to explore this consideration. The finding that female nonsmokers rated their competence in tobacco cessation counseling significantly lower than their male colleagues does not reflect actual differences but rather a possible negatively distorted self-perception towards reality [56-59]. Adequate training may help overcome this misperception and increase female medical students' self-confidence in their ability to provide smoking cessation advice or counseling.

The current study is subject to certain methodological limitations. First, our sample only consists of fifth-year medical students. Therefore, a comparison between students in preclinical and clinical years regarding smoking habits, smoking-related knowledge and students' perceived competence was not possible. Second, smoking status of subjects was assessed only by means of self-report, potentially rendering our results less reliable. However, the use of confirmatory carbon monoxide or cotinine tests was impracticable for such a large sample. Because the survey was anonymous and completely voluntary, one can assume that smoking status was reliably captured. Third, the design of our study was cross-sectional and this form of research can only provide a snapshot of the situation in the sample.

Nevertheless, the results of our study support the findings of Raupach and colleagues [20] and indicate an urgent need to better equip medical students to treat smoking patients. One way to counteract their insufficient knowledge is to provide adequate education in the medical curriculum, especially because medical school is an ideal time for training in smoking cessation techniques [60]. Roche and colleagues demonstrated significantly improved skills of medical students in smoking intervention after such training. This effect was not dependent on the mode of delivery [61]. Smoking-related knowledge of medical students in Hong Kong increased after a three hour seminar on tobacco [62].

Research suggests that role-playing, computer-assisted instructions, group discussions [63], and simulated patients [64] are useful methods in developing smoking cessation intervention skills. For this reason, a tobacco module should be integrated into the curriculum of every medical school, thus providing medical professionals with universal training in nicotine dependence intervention and smokers with healthcare professionals skilled to adequately assist them in their quit attempt.

## Competing interests

Tobias Raupach was reimbursed for attendance at two workshops on smoking cessation funded by Pfizer from 2006 through 2008. None of the other authors has any competing interests to declare

## Authors' contributions

TR, BK, and DAG conceived and designed the study. BK managed the data assessment. BK analyzed the data. BK wrote the manuscript. BK, DQ, KV, TW, SM, AMF, DAG and TR interpreted the data and contributed substantially to its revision. All authors read and approved the final manuscript.
